# Nucleocytoplasmic Shuttling Activity of Ataxin-3

**DOI:** 10.1371/journal.pone.0005834

**Published:** 2009-06-08

**Authors:** Sandra Macedo-Ribeiro, Luísa Cortes, Patrícia Maciel, Ana Luísa Carvalho

**Affiliations:** 1 IBMC-Instituto de Biologia Molecular e Celular, Universidade do Porto, Porto, Portugal; 2 Center for Neuroscience and Cell Biology (CNC), University of Coimbra, Coimbra, Portugal; 3 Life and Health Sciences Research Institute (ICVS), School of Health Sciences, University of Minho, Braga, Portugal; 4 Department of Zoology, University of Coimbra, Coimbra, Portugal; University of Cambridge, United Kingdom

## Abstract

Spinocerebellar ataxia type-3, also known as Machado-Joseph Disease (MJD), is one of many inherited neurodegenerative disorders caused by polyglutamine-encoding CAG repeat expansions in otherwise unrelated genes. Disease protein misfolding and aggregation, often within the nucleus of affected neurons, characterize polyglutamine disorders. Several evidences have implicated the nucleus as the primary site of pathogenesis for MJD. However, the molecular determinants for the nucleocytoplasmic transport of human ataxin-3 (Atx3), the protein which is mutated in patients with MJD, are not characterized.

In order to characterize the nuclear shuttling activity of Atx3, we performed yeast nuclear import assays and found that Atx3 is actively imported into the nucleus, by means of a classical nuclear localizing sequence formed by a cluster of lysine and arginine residues. On the other hand, when active nuclear export was inhibited using leptomycin B, a specific inhibitor of the nuclear export receptor CRM1, both endogenous Atx3 and transfected GFP-Atx3 accumulated inside the nucleus of a subpopulation of COS-7 cells, whereas both proteins are normally predominant in the cytoplasm.

Additionally, using a Rev(1.4)-GFP nuclear export assay, we performed an extensive analysis of six putative aliphatic nuclear export motifs identified in Atx3 amino acid sequence. Although none of the tested peptide sequences were found to drive nuclear export when isolated, we have successfully mapped the region of Atx3 responsible for its CRM1-independent nuclear export activity. Curiously, the N-terminal Josephin domain alone is exported into the cytoplasm, but the nuclear export activity of Atx3 is significantly enhanced in a longer construct that is truncated after the two ubiquitin interaction motifs, upstream from the polyQ tract.

Our data show that Atx3 is actively imported to and exported from the cell nucleus, and that its nuclear export activity is dependent on a motif located at its N-terminal region. Since pathological Atx3 aggregates in the nucleus of affected neurons in MJD, and there is *in vivo* evidence that nuclear localization of Atx3 is required for the manifestation of symptoms in MJD, defects in the nucleocytoplasmic shuttling activity of the protein may be involved in the nuclear accumulation and aggregation of expanded Atx3.

## Introduction

Machado-Joseph disease (MJD), or spinocerebellar ataxia type 3, is the most common dominantly inherited ataxia worldwide and it is caused by a polyglutamine (polyQ) expansion in ataxin-3 (Atx3), a polyubiquitin-binding protein [1] with ubiquitin protease activity [2]. Full-length Atx3 contains an N-terminal Josephin domain (JD), the conserved catalytic module, two ubiquitin interacting motifs (UIMs), an expandable polyQ stretch, and a short variable tail that might contain a third UIM depending on the splice variant [3]. In normal individuals the size of the polymorphic glutamine repeat can range between 14 and 40 while in MJD patients the polyQ repeat is expanded to 53 or more glutamines [4]. Human Atx3 is ubiquitously expressed and displays a complex subcellular distribution involving both the cytoplasm and the nucleus, depending on cell type [5], [6], [7]. Even though its physiological role is still not clearly established, Atx3 was shown to be a cysteine protease with the ability to cleave polyubiquitin chains with more than four ubiquitins, independently of the polyglutamine tract [2]. This enzymatic activity of Atx3 has been correlated with its ability to mitigate polyQ-induced neurodegeneration in a *Drosophila* model [8], with its involvement in aggresome formation [9] and in the degradation of misfolded proteins [10]. Furthermore, it was also shown to bind to histones [11] and to chromatin [12] indicating that Atx3 displays not only cytoplasmic but also nuclear functions.

As for most other polyQ diseases, conformational changes imparted by the expanded polyQ tract lead to the formation of neuronal intranuclear inclusions (NIIs) [13] and may contribute to pathogenesis by affecting gene expression [14] or by disrupting nuclear organization and function [15]. In MJD patients specific brain regions are affected such as the cerebellum and brainstem, with prominent cell loss in the pontine and dentate nuclei [6], [16], [17]. It is becoming clear that although polyQ tracts themselves are toxic, the sequence and structure of the proteins carrying the polyQ tracts have important roles in defining the course and specificity of the disease. Those sequences determine subcellular localization, and specify interactions with other macromolecules within the cell, strongly determining the differences in the specificity of neuronal degeneration characteristic of polyQ disorders [18], [19], [20].

A contentious question has been whether polyQ-induced pathogenesis is primarily activated in the cytoplasm or in the cell nucleus. In fact predominantly nuclear inclusions have been found in SCA1, SCA2, SCA7, SCA17, DRPLA, SBMA, and Huntington's Disease (HD) patients [21], although cytoplasmic inclusions have also been identified in affected brain regions in SCA2 [22] and HD [23]. Evidence from HD transgenic mice shows that both nuclear and cytoplasmic exon-1 huntingtin might contribute to disease progression [23], [24], [25]. Nuclear environment has been shown to favor toxicity, pathology and aggregation as evidenced by nuclear targeting of polyQ peptides [26], even when inserted into ectopic protein contexts [27].

Specific nuclear localization sequences (NLS) have been identified in proteins carrying the expanded polyQ tracts, such as ataxin-1 [28], [29] and ataxin-7 [30], and nuclear-associated mechanisms are being implicated in neuropathogenesis [13], [31]. Similarly, nuclear export sequences (NES) have been found in ataxin-7 [32] and huntingtin [33] and it was shown that polyQ expansion impairs efficient nuclear export of these polyQ-containing proteins [32], [34].

Recently, it was demonstrated *in vivo* that adding an exogenous NLS to Atx3(148Q) increases the severity of the phenotype and induces earlier death in transgenic mouse models [35]. Accordingly, adding an exogenous NES to Atx3(148Q) drives the expanded protein out of the nucleus and prevents the manifestation of a phenotype [35]. This suggests that defects on the nucleocytoplasmic shuttling activity of the expanded protein might be correlated with pathology and neuronal specificity. Moreover, in other transgenic models of Machado-Joseph disease there is accumulation of the expanded Atx3 protein in the nucleus of affected neurons [36], [37], [38]. However, the molecular determinants for the nucleocytoplasmic transport of Atx3 are not characterized. In order to gain further insights into the function of Atx3 and into the disease-specific mechanisms of neurodegeneration in MJD, we have set as our goal the identification of the determinants of Atx3 nucleocytoplasmic transport.

Nuclear targeting was analyzed *in vivo* using the yeast system developed by Rhee *et al.* [39] and we found that Atx3 is actively imported into the nucleus, by means of a classical NLS located in its C-terminal region. Furthermore, using an *in vivo* nuclear export assay we show that Atx3 is actively exported from the nucleus and mapped this export activity to its N-terminal domain.

## Methods

### Yeast nuclear import assay

The yeast nuclear import assay was performed as described previously [39]. The cDNA encoding the MJD1.a isoform of ataxin-3 (AAB33571), containing 28 CAG repeats, was amplified by PCR using the pEGFP-C1-ataxin-3(28Q) construct (kindly provided by Dr. Henry Paulson) as template and using the primers 5′tcccccgggcatggtgagcaagggcg3′ and 5′gcgtcgacttatgtcagataaagtgtgaag3′, which introduced SmaI and SalI recognition sites at the 5′ and 3′ ends of the amplified DNA fragment, respectively. The cDNA encoding ataxin-3(28Q) was cloned into the SmaI and SalI sites of the plasmid for the yeast nuclear import assay (pNIA, kindly provided by Vitaly Citovsky [39]), and named pNIA-GFP-Atx3. The pNIA-GFP-Atx3R282T and pNIA-GFP-Atx3R282A constructs were obtained by site-directed mutagenesis (QuickChange site-directed mutagenesis kit, Stratagene).

The pNIA constructs, encoding triple fusion proteins comprising bacterial LexA, yeast Gal4p activation domain (Gal4AD), and the tested protein, were transformed using the lithium acetate method [40] into *Saccharomyces cerevisiae* L40 strain, which contains the reporter genes *HIS3* and *lacZ* with upstream LexA operators. After transformation, yeasts were plated on selective medium deficient for tryptophan, to select for transformed cells. Transformed yeasts were then plated on selective medium lacking both tryptophan and histidine, and supplemented with 100 mM 3-amino-1,2,4-triazole (3AT; Sigma), an inhibitor of the His3p enzyme, and growth was evaluated. Additionally, transformed yeasts were grown in tryptophan-deficient liquid medium for quantitative determination of β-galactosidase activity [41]. For the enzymatic assay, cells were disrupted, and the β -galactosidase substrate *o*-nitrophenyl-β-*D*-galactopyranoside (ONPG) was added in excess. The reaction occurred at 30°C and was stopped by raising the pH to 11. The optical density of the reaction product was measured at 420 nm (OD420), and the β-galactosidase activity was calculated according to the following equation: βunits = 1,000×OD420/t×V×OD600, where OD420 is the optical density at 420 nm of the sample measured after the incubation of yeast cell lysate with ONPG, t is the time of incubation (in minutes) of the yeast cell lysate with ONPG, V is the volume of the sample used in the assay (in milliliters), and OD600 is the optical density at 600 nm of the yeast cell culture at the start of the assay.

### Cell culture, transfection and leptomycin B treatment

HEK293 and COS-7 cells were grown and maintained in Dulbecco's modified Eagle's medium-high glucose (DMEM-HG; Sigma) supplemented with 10% (vol/vol) heat-inactivated fetal bovine serum (FBS; Biochrom KG) and with 100 U of penicillin and 100 µg of streptomycin (Sigma) per ml in a 5% CO_2_ humidified atmosphere at 37°C.

One day prior to transfection, COS-7 cells were seeded onto glass coverslips on a 12-well plate, at a subconfluent density. Transfection experiments were performed using Lipofectamine reagent (Invitrogen) according to the manufacturer's instructions, using 1 µg of plasmid DNA per well. The cells were incubated for 48 hours to allow gene expression.

Where indicated, the cells were incubated with 20 ng/ml leptomycin B (Sigma) in DMEM-HG supplemented with 10% FBS for 3 hours prior to fixation.

### Rev(1.4)-GFP nuclear export assay

To assess the strength of the nuclear export activity of different fragments of Atx3 protein, or of full-length Atx3, the Rev(1.4)-GFP nuclear export assay, described previously by Henderson and Eleftheriou [42], was used. This assay is based on the manipulation of the Rev protein shuttling cycle, and tests for the ability of functional nuclear export sequences to promote the nuclear export activity of the Rev(1.4)-GFP fusion protein, which is composed of a NES-deficient mutant of the HIV-1 Rev protein and GFP.

Potential Atx3 NES signals were identified by consensus to the ΦX_1–3_ΦX_2–3_ΦXΦ motif (Φ indicates a large hydrophobic residue, and X indicates any amino acid), and using the prediction algorithm NetNES (http://www.cbs.dtu.dk/databases/NESbase-1.0 [43]), and fused to Rev(1.4)-GFP fusion protein (the plasmid encoding Rev(1.4)-GFP was kindly provided by Beric R. Henderson). The putative sequences that were assayed include: NES1, residues 76-SIQVISNALKVWGLELILF-94; NES2, 133-LNSLLT-138; NES3, 142-LISDTYLALFLAQLQQE-158; NES4, 174-ADQLLQMIRV-183; NES5, 209-LERVLE-214; and NES6, 222-LDEDEEDLQRALALSRQEIDME-243. Furthermore, full-length Atx3 (28Q and 84Q) or partial domains of Atx3 (the Josephin domain and the domains comprising amino acids 1–263 and 183–263) were also fused to Rev(1.4)-GFP fusion protein.

COS-7 cells were transfected with pRev(1.4)-GFP (negative control) or its derivative plasmids containing either the NES of the HIV-1 Rev protein (positive control) or each of the sequences to be tested. Forty-eight hours post-transfection, all cell samples were treated with 10 µg/ml cycloheximide (CHX) to ensure that any cytoplasmic GFP fluorescence resulted only from the nuclear export of GFP fusion proteins and not from *de novo* protein synthesis. Simultaneously, part of the cell samples were also treated for 3 hours with 5 µg/ml actinomycin D (ActD), which is known to specifically block the nuclear import of Rev protein by a mechanism not yet elucidated [42]. The remaining cell samples were treated with CHX, ActD and 20 ng/ml of leptomycin B for 3 hours. The subcellular localization of each GFP fusion protein was determined in at least 200 cells per experimental condition from three independent experiments.

### Fluorescence microscopy

Endogenous Atx3 was detected, by immunocytochemistry, with anti-MJD antibody (1∶10000), kindly provided by H. Paulson, and visualized using a secondary antibody labeled with Alexa 488 (1∶1000, Invitrogen). For fluorescence analysis of GFP fusion proteins, cells were washed with phosphate-buffered saline (PBS), fixed with 4% paraformaldehyde: 4% sacarose for 15 min, and rinsed with PBS. The coverslips were then inverted and mounted on glass slides with Vectashield mounting medium (Vector Laboratories). The cell nucleus was stained with Hoescht 33342 (0,5 µg/ml, Molecular Probes). Fluorescence observations were performed using a Zeiss Axiovert 200 fluorescence microscope, coupled to a digital photographic camera (Axiocam HRM). Confocal microscopy was performed using a Zeiss LSM 510 Meta system.

## Results and Discussion

### Ataxin-3 has a mixed cytoplasmic and nuclear distribution

**Figure 1:**
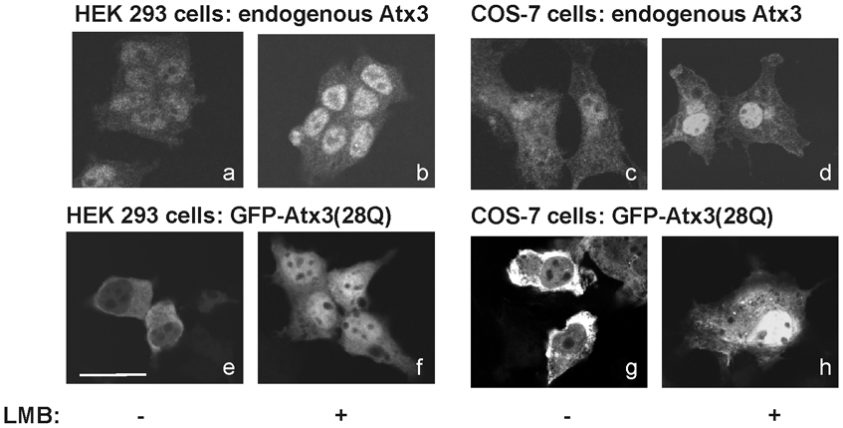
Ataxin-3 can shuttle between the nucleus and the cytoplasm. Endogenous Atx3 in HEK293T (a, b) and COS-7 (c, d) cell lines was detected by immunocytochemistry, using an anti-Atx3 antibody. Subconfluent cultures of HEK293 (e, f) and COS-7 cells (g, h) were transiently transfected with the plasmids encoding GFP-Atx3(28Q), and the localization of the fusion protein was detected by the GFP fluorescence. To explore the possibility of CRM1-mediated nuclear export of endogenous (b, d) or overexpressed (f, h) Atx3, cells were incubated with 20 ηg/ml of Leptomycin B (LMB), for 3 hours, prior to fixation. The subcellular localization of Atx3 was visualized by fluorescence microscopy.

Ataxin-3 is an ubiquitous protein that is found both in the cytoplasm [44] and in the nucleus [5], [7]. However, upon expansion of the polyQ tract the protein forms insoluble inclusions predominantly located inside the nucleus of the affected cells [6]. Interestingly, the localization of the protein inside the cell is critically dependent on the cell type [5], [7]. Immunostaining of HEK293T cells with an anti-Atx3 antibody (kindly provided by H. Paulson [6]) showed that endogenous Atx3 is predominantly located in the nucleus (Fig. 1a), while its distribution is more homogeneous in COS-7 cells (Fig. 1c). Interestingly, when both cell lines were transiently transfected with Atx3 N-terminally tagged with GFP (GFP-Atx3 (28Q), Figs. 1e and 1g), Atx3 was found predominantly in the cytoplasm of both cell types, in agreement with previous data [5], [6], [45].

In order to determine whether Atx3 can be actively transported across the nuclear membrane we analyzed the effect of leptomycin B, a specific covalent inhibitor of the nuclear export factor CRM1/exportin [46], [47], on the subcellular distribution of both endogenous and overexpressed Atx3 in HEK293 and COS-7 cell lines. In eukaryotic cells, nuclear export of proteins is frequently mediated by this nuclear export factor, which binds to nuclear export signals (NES) on cargo molecules. If Atx3 is a nuclear shuttling protein, interfering with its putative nuclear export would be expected to modify Atx3 subcellular localization by increasing the proportion of the protein localized in the nucleus. Indeed, after cell treatment with leptomycin B both endogenous Atx3 (Figs. 1b and f) and transfected GFP-Atx3 (28Q) (Figs. 1d, h) accumulate in the nucleus of a subpopulation of cells, suggesting that Atx3 exits the cell nucleus by active transport at least partially dependent on the CRM1/exportin pathway. In COS-7 cells the nuclear accumulation of GFP-Atx3(28Q) could be observed in 26.5±4.5% of cells after leptomycin B treatment, whereas in untreated cells only 4.8±2.4% of cells showed nuclear accumulation of the protein. Nuclear export dependent on CRM1 receptor has also been demonstrated for other proteins containing expandable polyglutamine tracts such as ataxin-7 [32] and huntingtin [33]. In agreement with what is observed for Atx3 in the context of the full-length protein, leptomycin B treatment of cultured cells tranfected with huntingtin lead to a partial nuclear accumulation of the protein corresponding to a 10% increase in nuclear fluorescence of huntingtin [33].

### Ataxin-3 contains a functional nuclear localization signal

**Figure 2:**
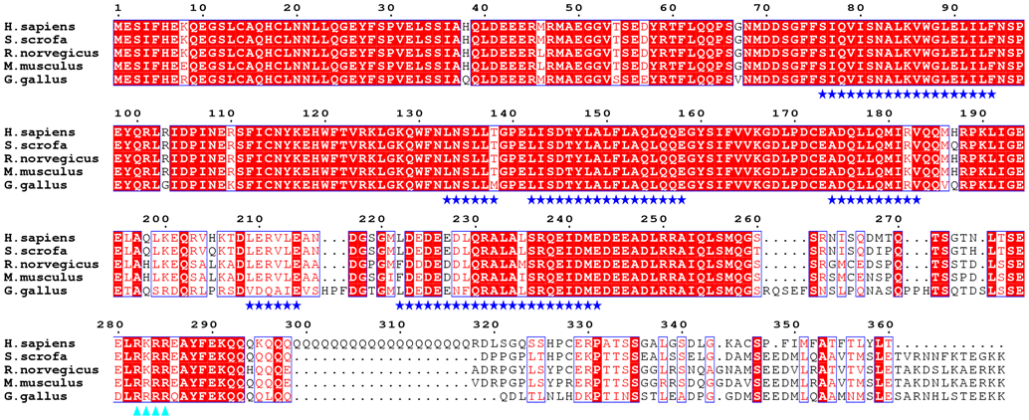
Human ataxin-3 and its closest homologues contain conserved nuclear import sequences. Sequence alignment of human (*H. sapiens*), wild boar (*S. scrofa*), mouse (*M. musculus*), rat (*R. norvegicus*), and chicken (*G. gallus*) ataxin-3. Identical residues are shown in white against a red background; conserved residues are shown in red. The conserved NLS sequence is highlighted by blue triangles below the alignment; the tested hydrophobic putative NES sequences are indicated by blue stars. The Josephin domain (JD) corresponds to residues 1 to 182, the UIM1 to residues 225 to 240 and the UIM2 to residues 246 to 259. The figure was prepared with ESPript [68].

Translocation of macromolecules larger than 40–60 kDa in and out of the nucleus is an active, energy-dependent process that is mediated by specific sequence motifs: nuclear localization signals (NLS) and nuclear export signals (NES). “Classical” examples of NLSs are the highly basic motifs originally found in the simian virus 40 (SV40) large T antigen and in nucleoplasmin, although several sequences differing from those basic ones have also been shown to function as NLS [48], [49], [50]. In “classical” nuclear import, importin-α recognizes and binds the target proteins in the cytoplasm, mediating their transport across the nuclear pore complex after formation of a ternary complex with importin-β [48], although some NLS-containing cargoes can be directly recognized by importin-β without the need of the adaptor protein. Within importin-α, the major NLS binding pocket contains a series of negatively charged residues that form salt bridges with the basic residues within the NLS sequences [51]. A putative NLS has been identified within the amino acid sequence of Atx3 [5], which is conserved among Atx3 proteins from diverse species (Fig. 2).

In order to test the functionality of the identified NLS sequence, we used a pNIA vector-based genetic one-hybrid system that allows identification of nuclear proteins in yeast cells [39]. This assay is based on the functional outcome of the nuclear import of the tested protein, which, if it reaches the yeast cell nucleus, allows specific induction of a reporter gene. The advantage of this approach is allowing the quantitative evaluation of the strength of the nucleocytoplasmic shuttling signals, independently of the cellular model. This system has been consistently used for mutational analysis of nuclear proteins to delineate and characterize the NLS [52] and for testing the integrity of the nuclear pore complex permeability barrier [53]. The rationale for this assay is the expression in the yeast *S. cerevisiae* cells of the test protein fused to a modified LexA DNA binding domain (DBD) and the Gal4 transcriptional activation domain (AD). This transcription based assay relies on the ability of a functional NLS to allow the chimera to enter the yeast nucleus and activate transcription of a LexA responsive β-galactosidase or *HIS3* reporter gene. In the absence of a functional NLS, the fusion protein is not efficiently imported into the nucleus, and is unable to activate transcription. As a result, this assay provides a simple measure of NLS function based either on the quantitative determination of β-galactosidase activity or on the qualitative analysis of yeast growth in a medium lacking histidine. This system is not limited to the identification of yeast NLSs, as the nuclear import apparatus is highly conserved between yeast and higher eukaryotic cells [49], [50].

**Figure 3:**
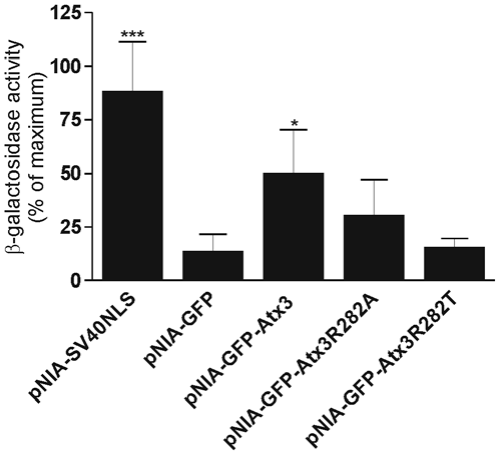
Evaluation of the nuclear import capacity of ataxin-3 protein in yeast. The β-galactosidase activity was quantified in liquid cultures after yeast cell growth in minimal medium lacking tryptophan. The data are expressed as a percentage of the enzymatic activity obtained for yeast cultures transformed with pNIA-SV40NLS; standard deviations are shown based on quadruplicates of at least three independent experiments.

pNIA-GFP and pNIA-SV40NLS were used as controls for the nuclear import assay. pNIA-GFP encodes the fusion protein mLexA-Gal4AD-GFP and was used as negative control because it is not imported into the nucleus, resulting in minimal expression of the two reporter genes, whereas pNIA-SV40NLS encodes the fusion protein mLexA-SV40NLS-Gal4AD-GFP which, due to the presence of the SV40NLS, is actively imported into the nucleus, leading to high activity of both lacZ and HIS3 genes. The cDNA for ataxin-3 (with 28 glutamines) was cloned in the pNIA vector, and to ensure that the resulting fusion protein had a molecular weight not compatible with simple diffusion across the nuclear pore, GFP was inserted between Gal4AD and Atx3. When expressed in yeast, the pNIA-GFP-Atx3 fusion protein induced growth on histidine-deficient medium (data not shown), suggesting that Atx3 was actively imported into the nucleus. To confirm this result we performed a quantitative β-galactosidase assay, in liquid culture of yeast cells expressing the different constructs (Fig. 3). The results obtained show that pNIA-GFPAtx3 induced levels of β-galactosidase activity significantly higher than the levels obtained for pNIA-GFP, a finding which confirms that Atx3 is actively imported into the nucleus of yeast cells.

To determine whether the proposed NLS of Atx3 was indeed responsible for the translocation of the fusion protein into the nucleus of yeast cells, we mutated the conserved arginine residue within the putative NLS sequence into a threonine residue (pNIA-GFP-Atx3R282T). This mutation is a typical mutation performed in the analysis of conserved NLS sequences since it changes a basic residue for a neutral residue without modifying its polar character [28], and has also been shown to disrupt the function of the NLS identified in ataxin-1 and ataxin-7 [28], [30]. As shown in Figure 3, the mutation results in the reduction the β-galactosidase activity to levels similar to the negative control (pNIA-GFP), indicating that the mutation greatly impairs Atx3 nuclear import in yeast. The mutation of the same arginine residue to an alanine residue (pNIA-GFP-Atx3R282A) was also tested, and resulted in a reduction on the nuclear accumulation of the fusion protein (Fig. 3). These data show that interference with the putative NLS disrupts the nuclear import ability of Atx3.

**Figure 4:**
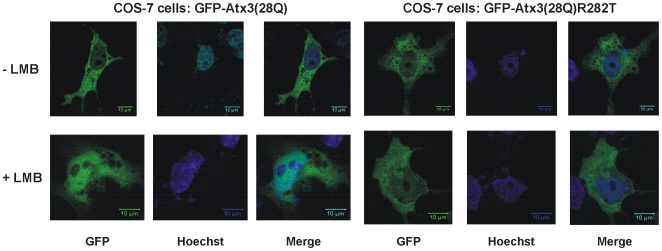
Evaluation of the nuclear import ability of ataxin-3 in mammalian cells. COS-7 cells expressing either GFP-Atx3(28Q), or GFP-Atx3(28Q)R282T, were analyzed by confocal fluorescence microscopy 48 h postransfection. GFP-Atx3(28Q) and GFP-Atx3(28Q)R282T are predominantly localized in the cytoplasm. Although the incubation with LMB (20 ng/ml, 3 h) lead to nuclear accumulation of GFP-Atx3(28Q), in 26.5±4.5% of the cells, LMB had no effect on GFP-Atx3(28Q)R282T subcellular localization. The cell nucleus was stained with Hoechst 33342.

The nuclear import activity of Atx3 was further confirmed in mammalian cells by comparing the subcellular localization of GFP-Atx3(28Q)R282T with GFP-Atx3(28Q) (Fig. 4). When transfected in COS-7 cells, both constructs localized mainly in the cytoplasm of the cells. Because our data show that Atx3 can also be exported from the nucleus and that this export is at least partially mediated by the CRM1 pathway (see above), the similar localization of wild-type Atx3 and the R282T mutant might be due to the presence of competitive nuclear export signals. Therefore, in order to investigate if there is a difference between the nuclear shuttling ability of the wild-type protein and the R232T mutant in COS-7 cells, we incubated the cells with leptomycin B, thereby at least partially inhibiting nuclear export, and determined their subcellular re-localization. As shown in Fig. 1 (panels g and h), GFP-Atx3(28Q) accumulates in the nucleus of a subpopulation of cells in the presence of leptomycin B. When GFP-Atx3(28Q)R282T is expressed in COS-7 cells this nuclear accumulation is not observed (Fig. 4), indicating that the identified NLS sequence is also responsible for driving Atx3 into the nucleus of mammalian cells. Therefore, we conclude that Atx3 contains a basic NLS sequence that is functional and promotes its active import into the cell nucleus.

### The nuclear export of ataxin-3 is mediated by CRM1-dependent and -independent pathways

**Figure 5:**
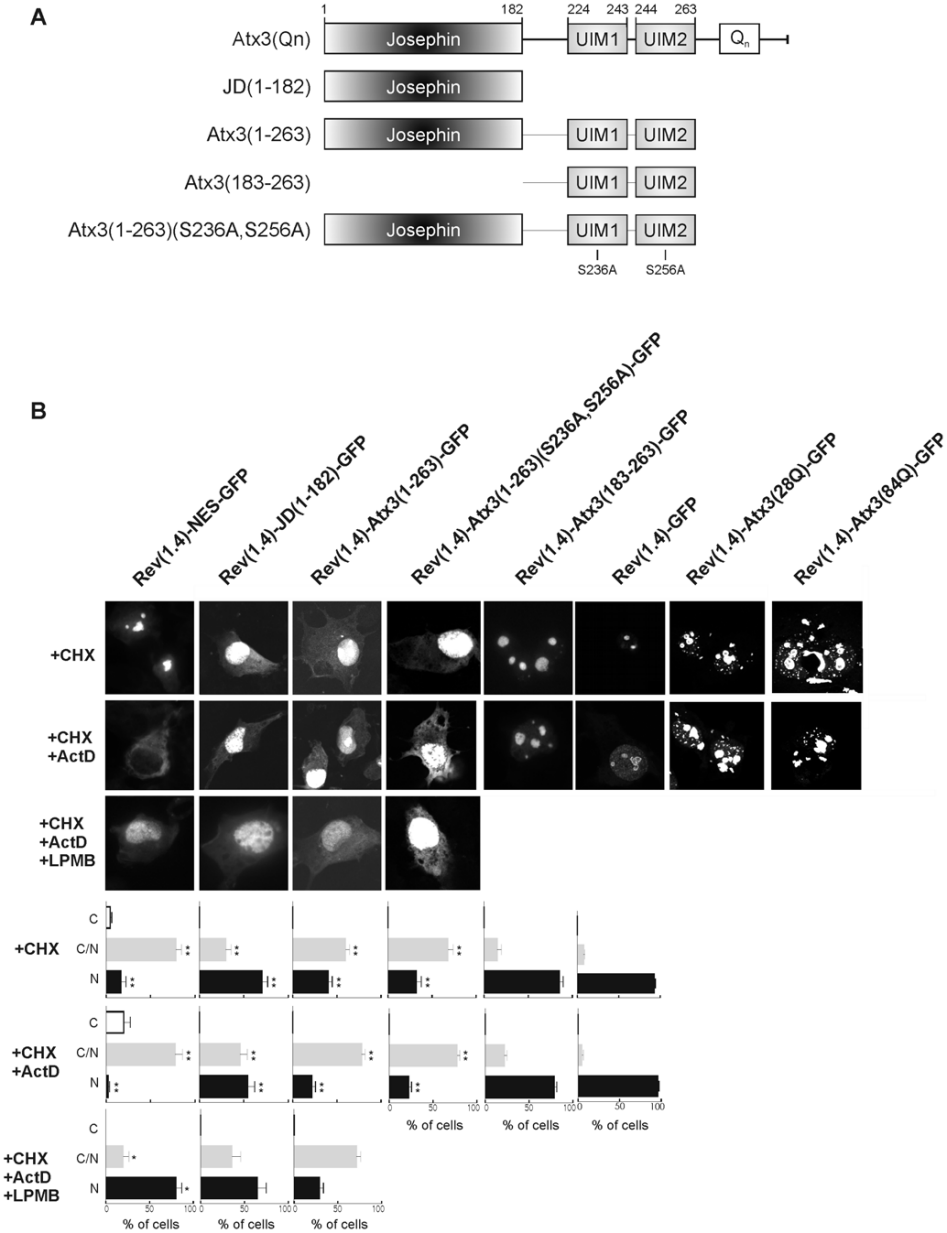
The nuclear export of ataxin-3 is mediated by the protein N-terminal domain. (a) Schematic representation of human Atx3, and tested Atx3 constructs used in the nuclear export assays in COS-7 cells. (b) Full-length Atx3 with 28 or 84 glutamine residues, as well as different Atx3 domains, were fused to an export-deficient mutant of Rev [Rev(1.4)-GFP], and their subcellular distributions were analyzed in transfected COS-7 cells. Rev(1.4)-NES–GFP, which contains the HIV-1 Rev NES, was used as a positive control. Forty-eight hours after transfection, cells were incubated for 3 hours with 10 µg/ml cycloheximide only (+CHX), to stop protein synthesis, treated with cycloheximide and 5 µg/ml actinomycin D (+CHX; +ActD), to block protein synthesis and the nuclear import of the Rev protein, or treated with cycloheximide, actinomycin D and 20 ηg/ml leptomycin B (+CHX; +ActD; +LMB), to additionally block CRM1-mediated nuclear export. The subcellular distribution of the different Rev(1.4)-GFP fusion proteins was examined by fluorescence microscopy, and representative images are shown. The percentage of transfected cells displaying exclusively nuclear, nuclear and cytoplasmatic, or exclusively cytoplasmatic GFP fusion proteins is plotted for each experimental condition. C, exclusively cytoplasmic; N/C, nuclear and cytoplasmic; and N, exclusively nuclear. Each experiment was performed at least three times, and approximately 200 cells were counted per experimental condition for every experiment. Error bars indicate standard deviations. All tested proteins have similar expression levels (data not shown). Rev(1.4)-Atx3(28Q)-GFP and Rev(1.4)Atx3(84Q)-GFP aggregate in the nucleus of transfected COS-7 cells, in the absence or presence of ActD.

Since the nuclear export of Atx3 was partially inhibited in the presence of leptomycin B (Figs. 1, 4), we analyzed the nuclear export activity of Atx3 using the Rev(1.4)-GFP nuclear export assay [42]. This assay is based on the expression of fusion proteins consisting of Rev(1.4), an export-defective HIV-1 Rev protein mutant, the sequence to be tested, and GFP. The nuclear export functionality of the sequence to be tested is evaluated by analyzing its capacity to promote nuclear export of the Rev(1.4)-GFP fusion protein. The Rev(1.4)-GFP fusion protein, which was used as a negative control for this assay, was localized exclusively in the nucleus of COS-7 cells, presenting a clear nucleolar accumulation (Fig. 5b). Rev(1.4)-NES–GFP, a fusion protein that contains the Rev NES, which was used as a positive control for this assay, was localized in the nucleus and the cytoplasm of transfected cells (Fig. 5b). The number of cells presenting exclusively cytoplasmic localization was enhanced following actinomycin D (ActD) treatment (Fig. 5b), which blocks the nuclear import mediated by the Rev NLS [42], whereas the cytoplasmic localization of Rev(1.4)-NES-GFP was completely blocked by addition of leptomycin B (Fig. 5b), as expected for a protein whose nuclear export is dependent on CRM1.

Full-length Atx3 containing 28 glutamine residues [Atx3(28Q)], when fused to Rev(1.4)-GFP, was driven into the nucleus, where it formed punctuate structures resembling the insoluble nuclear inclusions (Fig. 5b). The morphology of these structures is clearly distinct from the nucleolar accumulation of fluorescence observed for Rev(1.4)-GFP (Fig. 5b). We also tested full-length Atx3 containing 84 glutamine residues [Atx3(84Q), Fig. 5b], which formed punctuate structures, presumably aggregates, in the nucleus of transfected cells. These structures were not sensitive to the blockade of nuclear import of the Rev fusion protein using ActD. In fact, this result is in agreement with previous observations that Atx3 has a tendency to oligomerize independently of the expanded polyQ tract [54], [55], [56] and that the nuclear environment promotes protein misfolding and aggregation [57]. Curiously, it was observed that overexpression of ataxin-7 in cell culture models induced the nuclear localization of the protein and formation of large protein “accumulations” in ∼30% of the cells, independently of the polyglutamine tract size [30].

The nuclear aggregation of full-length Atx3 when fused to Rev(1.4)-GFP, independently of the extent of the polyQ tract, presumably triggered by the strong Rev NLS, hampered the investigation of the nuclear export activity of full-length Atx3 using this system. Therefore, we prepared constructs encoding various domains of Atx3 fused to Rev(1.4)-GFP, all lacking the polyglutamine tract and the endogenous NLS (Fig. 5a). The fusion protein consisting of the Josephin domain of Atx3 [JD-(1–182)] fused to Rev(1.4)-GFP showed a distribution between the cell nucleus and cytoplasm in 46% of the cells after cell treatment with ActD (Fig. 5b). We also tested the cellular localization of the fusion protein consisting of the Josephin domain followed by the segment of Atx3 that contains the UIMs [Atx3(1–263)], fused to Rev(1.4)-GFP [Rev1.4-Atx3(1–263)-GFP]. This protein also displayed a nuclear and cytoplasmic localization, and the number of cells showing a mixed distribution of the protein increased to 78% after cell treatment with ActD (Fig. 5b). The nuclear export activities of JD(1–182) and Atx3(1–263) were not inhibited by the CRM1 export inhibitor leptomycin B (+LMB).

These results suggest that the Josephin domain can mediate the nuclear export of the fusion proteins, which requires the context of the Josephin domain plus a sequence downstream this catalytic region. However, this UIM-containing segment alone [Rev(1.4)-Atx3(183–263)-GFP, Fig. 5b] was not sufficient to promote nuclear export of the fusion protein. Finally, we asked whether the nuclear export of the Atx3(1–263) fragment was dependent on the ubiquitin binding capacity of its UIMs. It has been shown, in a cell-based assay of polyQ aggregation, that recruitment of non-expanded Atx3 into nuclear aggregates is mediated by its UIMs [58]. Therefore, we mutated the two UIMs in the Atx3(1–263) fragment in order to compromise their functionality [Atx3(1–263)(S236A,S256A)]. This protein had the same pattern of cellular distribution as the wild-type Atx3(1–263) fragment (Fig. 5b), indicating that functionality of the UIMs is not required for the increased nuclear export activity observed for this region of Atx3.

Our results clearly demonstrate that nuclear export of Atx3 is dependent on a complex Atx3 motif, located in the N-terminal portion of the protein, which requires the context of the Josephin domain plus the ubiquitin-interacting motifs. Taken together, these data indicate either that nuclear export of Atx3 is mediated by a nuclear export receptor that recognizes a properly folded conformational motif and/or that multiple export pathways contribute to the overall nuclear export of full-length Atx3. Interestingly, a nuclear export receptor, exportin 7, has been recently described, which recognizes nuclear export signals that include conformation-dependent recognition motifs, rather than short linear sequences [59].

Since we observed nuclear accumulation of Atx3 in a population of COS-7 cells when the CRM1 transporter was inhibited with leptomycin B (Figs. 1 and 4), we looked for leucine-rich nuclear export signal (NES) sequences within Atx3 primary structure that might match the consensus NES sequence for the CRM1-dependent export [60], using the NetNES predictor (http://www.cbs.dtu.dk/databases/NESbase-1.0 [43]). We found 6 putative NES sequences, of which 4 are present within the Josephin domain (Fig. 2, NES1-NES4), one is located between the Josephin domain and the first ubiquitin interaction domain (NES5) and the last one (NES6) corresponds to the first ubiquitin interaction motif of the protein (Fig. 2). We have fused the putative NESs identified in Atx3 with Rev(1.4)-GFP, and tested whether the presence of these sequences can induce the translocation of Rev(1.4)-GFP from the cell nucleus. However, the isolated NES sequences failed to induce nuclear export when inserted into the Rev(1.4)-GFP vector (Table 1). The observation that in this system none of the tested sequences showed nuclear export activity can result from the fact that these sequences do not function as active leucine-rich nuclear export signals, but could also be a consequence of testing the sequences isolated from their overall protein context. For example, the isolated NES of Rev binds to CRM1 much more weakly than does the full-length Rev protein [61], implying that an NES may require flanking sequences to adopt the conformation needed for CRM1 binding. Another possibility is that each isolated sequence is not strong enough to drive detectable nuclear export of Rev1.4-GFP, which has a very strong NLS, and that several NESs work in concert to achieve efficient export of Atx3. In fact, recent studies show that most NESs bind to CRM1 with relatively low affinity, since high-affinity NES binding to CRM1 impairs the efficient release of export complexes from the nuclear pore complex [60].

**Table 1:** Analysis of the nuclear export activity of putative NES sequences within ataxin-3 primary structure.

Putative leucine-rich NESs within ataxin-3 (identified using NetNES)	Nuclear export activity in the Rev(1.4)-GFP nuclear export assay
NES1: 76 SIQVISNA**L**K**V**WG**L**E**LIL**F 94	-
NES2: 133 **L**NS**LL**T 138	-
NES3: 142 **LI**SDTY**L**A**L**F**L**AQ**L**QQE 158	-
NES4: 174 ADQ**LL**QM**I**R**V** 183	-
NES5: 209 **L**ERV**L** 214	-
NES6: 222 **L**DEDEED**L**QRA**L**A**L**SRQE**I**DME 243	-

Aliphatic residues fitting the consensus NES sequence ΦX_1–3_ΦX_2–3_ΦXΦ (where Φ indicates a large hydrophobic residue, and X indicates any amino acid) are highlighted in bold. The tested putative NESs in Atx3 did not show detectable nuclear export activity, as assessed using the Rev(1.4)-GFP nuclear export assay.

The nuclear export of GFP-Atx3(28Q) was partially inhibited by leptomycin B (Figs. 1, 4), suggesting that a CRM1-dependent pathway is involved in the nuclear export of Atx3. On the other hand, using the Rev(1.4)-GFP fusion system, which contains a strong NLS, we detected nuclear export activity of the N-terminal portion of Atx3 (Josephin domain and UIMs) that was not sensitive to leptomycin B (Fig. 5b). This supports the possibility that, in resemblance to what has been found for huntingtin [33], [34], CRM1-dependent and -independent nuclear export mechanisms coexist to cooperate in determining the subcellular distribution of Atx3. In the context of the full-length protein, one of these pathways may be dominant when compared to the other. Similar to that of Atx3 and huntingtin, the nuclear export of other proteins, such as α-catenin [62], receptor-interacting protein 3 (RIP3) [63] and the African Swine Fever Virus p37 protein [64], has been shown to be mediated by both CRM1-dependent and –independent pathways.

### Conclusion

A correlation between the nuclear environment and protein aggregation in MJD models has been pinpointed by studies using Atx3 with an exogenously added NLS that demonstrated *in situ* that the nuclear environment drives aggregation of both expanded and non-expanded Atx3 [57], [65]. Recent *in vivo* studies have shown that adding an exogenous NES does not only suppress the formation of NIIs almost completely, but also seems to prevent the aggregation of Atx3 [35]. Furthermore, live-cell imaging studies showed that expanded polyQ tracts slow the dynamics of intact Atx3, and that the export of expanded Atx3 is less efficient than the export of normal Atx3 [66], suggesting a defect on the nucleocytoplasmic shuttling activity of the expanded protein. In fact, a recent study using a Drosophila system to screen for genetic modifiers of Atx3 neurodegeneration identified as a suppressor of Atx3-mediated toxicity, among others, the gene encoding the nuclear export protein Embargoed, the orthologue of human CRM1 [67]. In order to further understand the link between nuclear localization, aggregation and toxicity of expanded Atx3, it is essential to identify the mechanisms behind the intracellular dynamics of the normal protein. In this study, we have confirmed the functionality of the putative Atx3 NLS in yeast and mammalian cells, and detected CRM1-dependent and –independent pathways that mediate the nuclear export of Atx3. Our data show that the CRM1-independent nuclear export of Atx3 is mediated by the N-terminal region of the protein and is critically dependent on a three-dimensional motif whose integrity is compromised when the Josephin domain is physically separated from the UIMs. Future identification of the nuclear transporter(s) responsible for this pathway, will pave the way to determining how the subcellular localization of Atx3 is regulated in physiological or pathological conditions.
